# Less renal allograft fibrosis with valganciclovir prophylaxis for cytomegalovirus compared to high-dose valacyclovir: a parallel group, open-label, randomized controlled trial

**DOI:** 10.1186/s12879-018-3493-y

**Published:** 2018-11-15

**Authors:** Tomas Reischig, Martin Kacer, Petra Hruba, Hana Hermanova, Ondrej Hes, Daniel Lysak, Stanislav Kormunda, Mirko Bouda

**Affiliations:** 1 Department of Internal Medicine I, Faculty of Medicine in Pilsen, Charles University, Czech Republic and Teaching Hospital, 30460 Pilsen, Czech Republic; 2 Biomedical Centre, Faculty of Medicine in Pilsen, Charles University, 32300 Pilsen, Czech Republic; 3 Transplant Laboratory, Institute for Clinical and Experimental Medicine, 14021 Prague, Czech Republic; 4 Department of Hemato-oncology, Teaching Hospital, 30460 Pilsen, Czech Republic; 5 Department of Pathology, Faculty of Medicine in Pilsen, Charles University, Czech Republic and Teaching Hospital, 30460 Pilsen, Czech Republic; 6 Division of Information Technologies and Statistics, Faculty of Medicine in Pilsen, Charles University, 32300 Pilsen, Czech Republic

**Keywords:** Cytomegalovirus, Valganciclovir, Prophylaxis, Fibrosis, Renal transplantation

## Abstract

**Background:**

Cytomegalovirus (CMV) prophylaxis may prevent CMV indirect effects in renal transplant recipients. This study aimed to compare the efficacy of valganciclovir and valacyclovir prophylaxis for CMV after renal transplantation with the focus on chronic histologic damage within the graft.

**Methods:**

From November 2007 through April 2012, adult renal transplant recipients were randomized, in an open-label, single-center study, at a 1:1 ratio to 3-month prophylaxis with valganciclovir (*n* = 60) or valacyclovir (*n* = 59). The primary endpoint was moderate-to-severe interstitial fibrosis and tubular atrophy assessed by protocol biopsy at 3 years evaluated by a single pathologist blinded to the study group. The analysis was conducted in an intention-to-treat population.

**Results:**

Among the 101 patients who had a protocol biopsy specimen available, the risk of moderate-to-severe interstitial fibrosis and tubular atrophy was significantly lower in those treated with valganciclovir (22% versus 34%; adjusted odds ratio, 0.31; 95% confidence interval, 0.11–0.90; *P* = 0.032 by multivariate logistic regression). The incidence of CMV disease (9% versus 2%; *P* = 0.115) and CMV DNAemia (36% versus 42%; *P* = 0.361) were not different at 3 years.

**Conclusions:**

Valganciclovir prophylaxis, as compared with valacyclovir, was associated with a reduced risk of moderate-to-severe interstitial fibrosis and tubular atrophy in patients after renal transplantation.

**Trial registration:**

Australian New Zealand Clinical Trials Registry (ACTRN12610000016033). Registered on September 26, 2007

**Electronic supplementary material:**

The online version of this article (10.1186/s12879-018-3493-y) contains supplementary material, which is available to authorized users.

## Background

Management of infectious diseases is a critical component of care of solid organ transplant recipients. Both cytomegalovirus (CMV) disease and asymptomatic CMV replication results in increased mortality and graft loss rates [1–5]. These impacts are driven mainly by the cellular and immunological effects of CMV including alloimmune response upregulation by innate immune mechanisms and/or cross-reactivity of CMV-specific T cells with donor MHC-peptide complexes [1, 6–9]. In addition to enhanced graft rejection rates, CMV has been shown to increase the risk of cardiovascular events and other opportunistic infections and is likely to play a role in the development of diabetes and cancer after transplantation [10–13].

Renal allograft fibrosis is the ultimate non-specific histological picture of various allograft injuries [14, 15]. Moderate-to-severe interstitial fibrosis and tubular atrophy (IFTA) is strong predictor of deteriorated graft survival [16]. The underlying mechanism in the development and progression of fibrosis is activation of the inflammatory cascade with subsequent formation of profibrotic mediators such as TGF-β [14]. Together with donor characteristics, there are a host of post-transplant contributors to IFTA development including inflammation associated with viral infections [14, 15]. Multiple studies have documented upregulation of profibrotic and vasculopathic growth factors in CMV infection, with some studies also reporting a higher incidence of IFTA in patients after previous CMV replication [6, 17–19].

CMV prevention taking the form of prophylaxis or preemptive therapy is recommended [2]. Either strategy results in a reduced incidence of CMV disease and infection, with lower mortality and acute rejection rates as an additional plus with prophylaxis [20–23]. As an alternative to valganciclovir prophylaxis in renal transplant recipients, valacyclovir has also been well documented to be effective [20, 22–24]. Still, in a long-term comparison with valganciclovir-based pre-emptive therapy, valacyclovir prophylaxis was associated with a higher incidence of severe IFTA and inferior graft survival [25].

To establish potential differences between valganciclovir and valacyclovir prophylaxis, we performed a randomized trial (2VAL Study) showing a decrease in the rates of acute rejection with valganciclovir [26]. This article presents the long-term results of the 2VAL Study focused primarily on the incidence of IFTA in late protocol biopsies.

## Methods

### Patients and interventions

Details of the study design were published previously [26]. In brief, adult renal transplant recipients from a single center were recruited from November 2007 through April 2012. The major exclusion criterion was recipient (R) and donor (D) negative CMV serology (D−/R-). Patients were randomized by the transplant physician using a random-number table, at a 1:1 ratio, to valganciclovir (900 mg daily) or valacyclovir (2 g four times daily) prophylaxis for 3 months. Sequentially numbered sealed envelopes were used for allocation concealment. Polymerase chain reaction (PCR) for CMV DNA from whole blood was performed at predefined time points during the first 12 months [26]. After 12 months, PCR was performed only if clinically required.

The protocol of immunosuppression was described previously [26]. Recipients of grafts from highly marginal donors were treated with basiliximab and low-dose tacrolimus. Polyoma BK virus (BKV) DNAemia was tested every month for the first 6 months, 3 months until 24 months, at 36 months, and if clinically indicated.

### Study outcomes and follow-up

The primary endpoint was the incidence of moderate-to-severe IFTA assessed on protocol biopsy at 36 months. Secondary endpoints included intrarenal mRNA expression of profibrotic genes, chronic rejection, CMV DNAemia, CMV disease, biopsy-proven acute rejection, renal function, patient and graft survival (not censored for death), and other infections. In addition, other potential indirect effects of CMV such as cardiovascular events or new-onset diabetes mellitus, malignancy, and routine laboratory parameters were recorded prospectively. All patients remained on follow-up for a minimum of 4 years after transplantation or until death. Patient and graft survival was assessed at 4 years, with other variables at the end of 3 years.

### Protocol biopsy sample processing

In patients with functioning grafts, protocol biopsy was performed at 36 months using an 18-gauge needle (biopsy gun). A minimum of two cores were obtained. Tissues for light microscopy were fixed in 4% formaldehyde, embedded in paraffin using routine procedure, and processed as described previously [25]. All biopsies were evaluated according to the Banff classification by a single pathologist blinded to the study group of the patients [27]. Intrarenal mRNA expression analysis was performed as described previously (Additional file 1: Table S1) [4, 25].

### Sample size and statistical analysis

We anticipated a 40% incidence of moderate-to-severe IFTA in late protocol biopsy [28]. Based on the association between acute rejection and IFTA [14, 15] and reduction in acute rejection with valacyclovir [23, 24], we assumed a 50% reduction in the relative risk for moderate-to-severe IFTA in the valacyclovir group. It was necessary to enroll at least 82 patients to ensure an 80% power to detect a treatment difference with a type 1 error of 0.05. A minimum of 114 patients was required for the 12-month primary endpoint (acute rejection) assessment [26]. This number was considered sufficient even with the anticipation of patients lost to late protocol biopsy.

Quantitative parametric data were compared using Student’s t-test and the Mann-Whitney U-test in non-parametric distribution. Qualitative data were analyzed using the chi-square or Fisher exact test. The risk of moderate-to-severe IFTA in the valganciclovir group compared to valacyclovir was calculated by logistic regression. Because of an imbalance in high-risk donor distribution and related immunosuppression, the odds ratio (OR), and 95% confidence interval (CI) adjusted for calcineurin inhibitor, induction therapy, and advanced chronic histologic damage (moderate-to-severe nephrosclerosis, diabetic nephropathy and/or ≥ 15% of glomerulosclerosis) in donor procurement biopsy were calculated by multivariate logistic regression. The incidence of time dependent variables was calculated using Kaplan-Meier curves, with the log-rank test and the Cox proportional hazard model adjusting for the above variables. Data were analyzed according to the intention-to-treat principle. Statistical calculations were made using SAS software (SAS Institute Inc., Cary, NC). Values of *P* < 0.05 were considered statistically significant.

## Results

### Study population

**Fig. 1:**
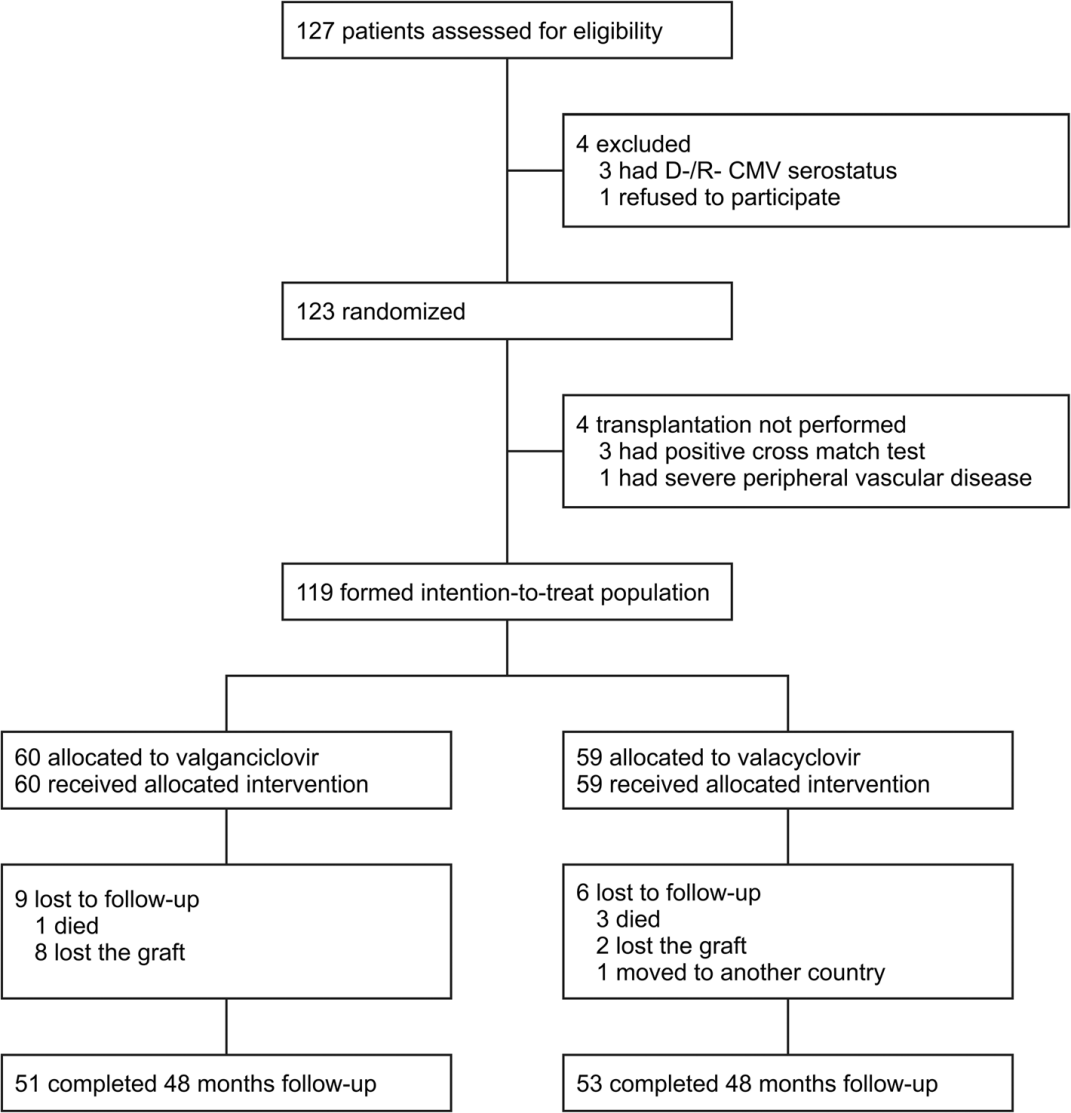
Flow of patients through the study. CMV, cytomegalovirus; D, donor; R, recipient

Overall, 119 patients were enrolled (Fig. 1), of which number 60 were randomized to valganciclovir prophylaxis and 59 to valacyclovir. The almost double the number of patients treated with basiliximab induction and low-dose tacrolimus protocol, indicated only in high-risk donors, and a detailed analysis of donor procurement biopsies revealed a lower quality of donors in the valganciclovir group (Table 1, Additional file 1: Table S2). The groups did not differ in maintenance immunosuppressive therapy including drug levels and doses in the ensuing years (Additional file 1: Table S3).

**Table 1:** Patient characteristics of the intention-to-treat population

Characteristic	Valganciclovir (*n* = 60)	Valacyclovir (*n* = 59)	*P* Value
Recipient			
Age (yr)	48 ± 13	50 ± 11	0.224
Gender (male)	47 (78)	37 (63)	0.095
Previous transplantation	9 (15)	7 (12)	0.816
HLA mismatches (n)	3.5 ± 1.2	3.6 ± 1.5	0.508
CMV serostatus			0.289
D+/R-	7 (12)	4 (7)	
D+/R+	44 (73)	49 (83)	
D−/R+	9 (15)	6 (10)	
Donor			
Age (yr)	50 ± 16	49 ± 16	0.702
Donor type (deceased)	57 (95)	54 (92)	0.696
Expanded-criteria donor^a^	34 (57)	32 (54)	0.935
Advanced chronic histologic damage^b^	15 (25)	9 (15)	0.185
Primary immunosuppression^c^			
Cyclosporine + mycophenolate mofetil	25 (42)	35 (59)	0.081
Tacrolimus + mycophenolate mofetil	35 (58)	24 (41)	
No induction therapy	25 (42)	34 (58)	0.119
Basiliximab	26 (43)	14 (24)	0.039
Thymoglobulin	9 (15)	11 (19)	0.775

Data are number of patients (percentage) or mean ± standard deviation. CMV, cytomegalovirus; D, donor; R, recipient

^a^According to the United Network for Organ Sharing criteria

^b^A minimum 1 of the following findings on donor procurement biopsy: moderate-to-severe vascular nephrosclerosis, diabetic nephropathy, and/or ≥ 15% of glomerulosclerosis. Procurement biopsy was performed in 61 selected donors considered to be at increased risk

^c^Low-dose tacrolimus with basiliximab induction was used in recipients of grafts from highly marginal donors (age ≥ 70 years, donors with hypertension or diabetes with impaired renal function or biopsy findings of vascular nephrosclerosis, diabetic nephropathy and/or ≥ 15% of glomerulosclerosis, donors after cardiac death, and dual kidney transplantation)

### Primary endpoint: IFTA in protocol biopsy at 3 years

Protocol biopsy at 36 months with sufficient material was performed in 51 and 50 patients in the valganciclovir and valacyclovir groups, respectively. The main causes for not performing biopsy were death or graft loss. Moderate-to-severe IFTA was less frequent in the valganciclovir group (11 of 51 [22%] versus 17 of 50 [34%]; OR, 0.53; 95% CI, 0.22–1.30; *P* = 0.166 by logistic regression) (Table 2). After adjustment for baseline characteristics, which reflected the higher proportion of high-risk donors in the valganciclovir group, the risk for developing moderate-to-severe IFTA was significantly lower in valganciclovir-treated patients (aOR, 0.31; 95% CI, 0.11–0.90; *P* = 0.032 by multivariate logistic regression). Advanced chronic histologic damage in donor procurement biopsy was significantly associated with moderate-to-severe IFTA (aOR, 7.05; 95% CI, 2.28–21.8; *P* < 0.001). While patients experiencing acute rejection showed a trend toward an increase in moderate-to-severe IFTA (41% versus 23%; aOR, 2.89; 95% CI, 0.88–9.73; *P* = 0.087), the effect of polyoma BKV viremia was negligible (29% versus 27%; aOR, 0.60; 95% CI, 0.18–2.07; *P* = 0.422).

**Table 2:** Histological findings in protocol biopsy at 36 months after transplantation

Characteristic	Valganciclovir (*n* = 51)^a^	Valacyclovir (*n* = 50)^a^	aOR (95% CI)^b^	*P* Value^c^
Glomeruli per biopsy	10.5 ± 7.6	13.0 ± 6.0		0.004
Arteries per biopsy	1.7 ± 0.9	1.7 ± 1.1		0.903
Moderate-to-severe IFTA^d^	11 (22)	17 (34)	0.31 (0.11–0.90)	0.032
IF/TA (all grades)	21 (41)	24 (48)		0.624
Chronic “ci + ct” score	1.64 ± 1.64	1.82 ± 1.59		0.529
Subclinical rejection	2 (4)	2 (4)		0.624
Borderline changes	7 (14)	3 (6)		0.334
Chronic antibody-mediated rejection	6 (12)	6 (12)		0.786
Chronic T-cell-mediated rejection	2 (4)	1 (2)		0.986
Calcineurin inhibitor toxicity	1 (2)	2 (4)		0.986
Vascular nephrosclerosis	13 (25)	14 (28)		0.952
Glomerulonephritis recurrence	2 (4)	0 (0)		0.484

Data are number of patients (percentage) or mean ± standard deviation. aOR, adjusted odds ratio; CI, confidence interval; IFTA, interstitial fibrosis and tubular atrophy; ci, interstitial fibrosis score; ct, tubular atrophy score

^a^Biopsy not available in valganciclovir: 6 death or graft loss, 1 refused, 2 insufficient material; in valacyclovir: 4 death or graft loss, 1 lost to follow up, 1 technical reason, 3 insufficient material

^b^Adjusted for calcineurin inhibitor, induction therapy, and advanced chronic histologic damage in donor biopsy

^c^Multivariate logistic regression for moderate-to-severe IFTA comparison; chi-squared or Fisher exact test for categorical variables; Mann-Whitney U-test for continuous variables

^d^Grade 2 or more according to the Banff 2013 classification

Patients with moderate-to-severe IFTA showed increased intrarenal mRNA expression of a wide range of profibrotic genes (Additional file 1: Table S4). However, univariate unadjusted analysis did not document any significant differences between the valganciclovir and valacyclovir groups (data not shown).

### CMV disease and DNAemia

**Fig. 2:**
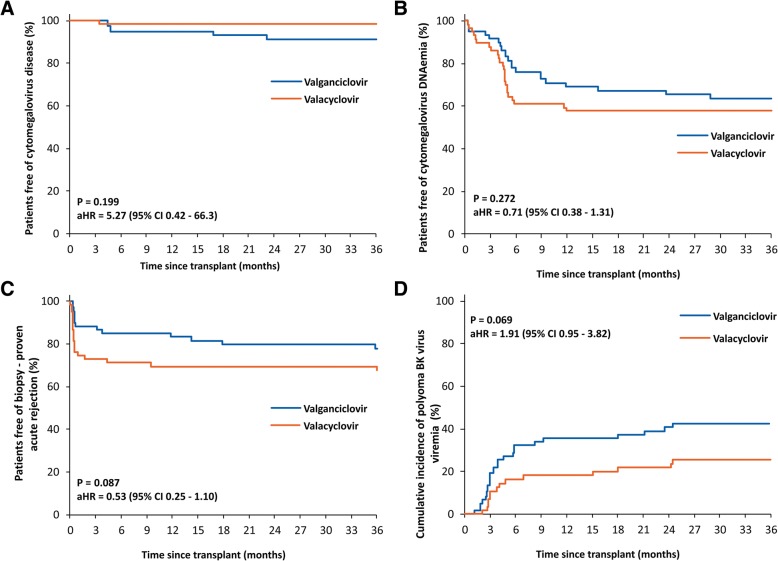
Kaplan-Meier curves for the cumulative probability of freedom from CMV disease (**a**), CMV DNAemia (**b**), biopsy-proven acute rejection (**c**), and polyoma BK virus viremia (**d**). The Cox proportional hazard model adjusting for calcineurin inhibitor, induction therapy, and advanced chronic histologic damage in donor biopsy. *aHR* adjusted hazard ratio, *CI* confidence interval

After 12 months, CMV disease was diagnosed in 2 valganciclovir group patients. One case involved CMV syndrome in a D+/R- patient, the other CMV colitis after thymoglobulin administration for acute rejection despite a new course of valganciclovir prophylaxis. The difference was not statistically significant at 36 months (Fig. 2a). After 12 months, CMV DNAemia was present in 5 patients in the valganciclovir group with 3 cases involved a new-onset episode and 2 a recurrent one in contrast to 1 recurrent episode in the valacyclovir group. CMV DNAemia with viral load of ≥1000 copies/mL comprised 3 out of 5 episodes in the valganciclovir group and a single episode in the valacyclovir group. At 36 months, the cumulative incidence of CMV DNAemia was comparable in both groups (Fig. 2b and Table 3).

**Table 3:** Details on CMV infection during 36 months

Characteristic	Valganciclovir (*n* = 60)	Valacyclovir (*n* = 59)	*P* Value
CMV Disease	5 (9)	1 (2)	0.115^a^
CMV Disease by D/R status			
D+/R-	3 (49)	0 (0)	0.125
D+/R+	2 (5)	1 (2)	0.555
D−/R+	0 (0)	0 (0)	–
CMV DNAemia	21 (36)	24 (42)	0.361^a^
CMV DNAemia by D/R status			
D+/R-	4 (64)	2 (50)	0.986
D+/R+	14 (32)	21 (45)	0.180
D−/R+	5 (50)	1 (17)	0.264
Peak viral load (copies/mL)	350 (100–6150)	850 (100–1650)	0.982
Duration of CMV DNAemia (d)	40 (27–78)	31 (15–69)	0.270

Data are number of patients (percentage) or median and interquartile range. CMV, cytomegalovirus; D, donor; R, recipient

^a^CMV Disease: adjusted hazard ratio, 4.96; 95% confidence interval, 0.57–43.1; *P* = 0.147; CMV DNAemia: adjusted hazard ratio, 0.78; 95% confidence interval, 0.43–1.42; *P* = 0.418 by multivariate Cox proportional hazard model after adjustment for calcineurin inhibitor, induction therapy, and advanced chronic histologic damage in donor biopsy

### Rejection, polyomavirus infection, and other outcomes

After 12 months, late acute rejection was detected in 4 and 1 patients in the valganciclovir and valacyclovir groups, respectively. In the valganciclovir group, the event was preceded, in all 4 patients, by their demonstrable noncompliance or immunosuppression reduction due to infectious complications (Fig. 2c).

While, after 12 months, a new-onset episode of polyoma BKV viremia was documented in 4 patients in both the valganciclovir and valacyclovir groups, the cumulative incidence remained – given the differences within the first 12 months – higher with valganciclovir (42% versus 26%; *P* = 0.046 by log-rank test) (Fig. 2d). Likewise, although not significantly different, the incidence of polyomavirus-associated nephropathy (PVAN) was higher in the valganciclovir group (12% versus 4%; *P* = 0.098 by log-rank test). PVAN had an appreciably adverse effect on graft survival (56% versus 91%; *P* < 0.001 by log-rank test) with 3 graft losses directly related to PVAN in valganciclovir-treated patients. The groups did not differ in the incidence of other viral, bacterial, and fungal infections.

At 4 years, patient and graft survival rates were excellent in either group. Regarding the other secondary outcomes, no differences were found between the groups (Table 4).

**Table 4:** Patient and graft survival and other secondary outcomes

Characteristic	Valganciclovir (*n* = 60)	Valacyclovir (*n* = 59)	*P* Value
Patient survival	59 (98)	56 (95)	0.297
Graft survival	51 (85)	54 (92)	0.287
Estimated GFR^a^ (mL/min/1.73 m^2^)	54 ± 20	52 ± 19	0.594
Urine protein-to-creatinine ratio (mg/mmol)	23 ± 49	24 ± 43	0.339
Cardiovascular events	15 (25)	14 (24)	0.985
New-onset diabetes or IFG	26 (46)	20 (38)	0.521
Malignancy	6 (10)	2 (3)	0.283

Data are number of patients (percentage) or mean ± standard deviation. GFR, glomerular filtration rate; IFG, impaired fasting glucose

^a^According to the Modification of Diet in Renal Disease 7 formula

## Discussion

Long-term results of this only randomized study comparing valganciclovir and valacyclovir for CMV prophylaxis in renal transplant recipients published to date demonstrated a lower incidence of moderate-to-severe IFTA at 3 years in patients treated with valganciclovir. This finding was unexpected at study initiation given the promising results of valacyclovir prophylaxis in reducing the risk of rejection in earlier studies [23, 24]. Still, it is fully consistent with the 12-month data of the present study documenting a significant decrease in acute rejection rates with valganciclovir prophylaxis. By contrast, polyoma BKV viremia, whose incidence was increased at 12 months in the valganciclovir group, had no adverse impact on the risk of IFTA [26]. Severe forms of IFTA are associated with a marked increase in the risk of graft failure [14, 16]. The negative effect of IFTA is further enhanced by co-existing graft inflammation or circulating donor-specific anti-HLA antibodies (DSA) [15, 16]. While effective prevention of IFTA from developing is critical for improving transplantation outcomes, it is most difficult because of its multifactorial etiology [14]. Our study has suggested that optimal CMV prevention could be part of a comprehensive strategy to reduce IFTA incidence.

The dominant factors leading to the development and progression of IFTA include nonspecific inflammation and, particularly, inflammation secondary to alloimmune activation. Acute rejection, subclinical rejection including antibody-mediated rejection or the presence of DSA have a strong profibrotic potential and result in the development of severe IFTA with overlapping gene expression profile of biopsies with IFTA and immune-mediated inflammation [15, 29–31]. Because of CMV-associated intragraft inflammation, CMV is a potential risk factor for IFTA regardless of whether the underlying mechanism is heterologous immunity or promotion of local inflammation [6]. Cytomegalovirus significantly increases the risk of acute cellular rejection and, in patients with DSA, it may be involved in the pathogenesis of antibody-mediated rejection [8, 10]. In our study, the reduction of IFTA in patients receiving valganciclovir prophylaxis cannot be explained by different efficacy in CMV prevention. The rates of CMV DNAemia were comparable both in the long-term and early period (within 3 months) after transplantation [26]. The most logical cause is the decrease in acute rejection in patients randomized to valganciclovir [26]. Consistent with the above studies, the risk of our patients experiencing acute rejection to develop moderate-to-severe IFTA was almost three times higher. The difference in the incidence of acute rejection occurred in the early post-transplant period. One could speculate that the incidence of subclinical inflammation over the first post-transplant weeks could be also decreased with valganciclovir. While, in our study, the first protocol biopsy was not performed until month 3, recent data suggest a high proportion of inflammation associated mainly with mild IFTA detected by protocol biopsy at 6 weeks [30]. Early subclinical inflammation constitutes a risk factor for IFTA progression [30].

The present study is a second trial documenting inferior outcomes of valacyclovir prophylaxis compared to valganciclovir-based CMV prevention. Compared with preemptive therapy, valacyclovir prophylaxis was associated with a higher incidence of IFTA and profibrotic gene expression [25]. In an animal model, ganciclovir prophylaxis attenuated late renal allograft damage and reduced intragraft immune infiltrates persisting even after prophylaxis discontinuation [32]. A theory explaining the lower rates of acute rejection and subsequent IFTA development with valganciclovir prophylaxis is built on reduced intragraft inflammation through a direct effect of ganciclovir on T-lymphocytes. In studies with healthy volunteers and renal transplant recipients, (val) ganciclovir suppressed T-lymphocyte proliferation and activation by impaired DNA synthesis [33, 34]. Further studies are warranted to confirm the clinical relevance of this theory.

The decrease in IFTA over the 4 years of follow-up did not result in better graft survival in the valganciclovir group. Lower quality donors may have influenced graft survival. While it is likely that follow-up may have been still too short in this respect, an alternative explanation may be an increased incidence of polyoma BKV infection. In our study, BKV viremia was not shown to negatively impact the risk of moderate-to-severe IFTA or graft loss, which comes as no surprise since low-viral load BKV viremia was predominantly involved. The risk of graft loss or graft dysfunction is increased with persistent high-viral load BKV viremia or PVAN, where recent data have additionally suggested increased de novo production of DSA [35, 36]. The numerically higher incidence of PVAN in patients treated with valganciclovir prophylaxis requires extra caution. While our study was not powered to detect significant differences in the incidence of PVAN, the rates were clinically not negligible with 3 cases of graft loss directly related to PVAN in the valganciclovir group. Also in a recently published study, full dose valganciclovir prophylaxis resulted in higher rates of PVAN [37]. It is conceivable that valganciclovir had an effect on the BKV-specific cellular immune response making BKV replication more frequent [38, 39].

Several limitations of the study should be mentioned. Despite adequate randomization, there was an imbalance in the proportion of high-risk donors tilted against the valganciclovir group. As expected, tacrolimus-based immunosuppression indicated in at-risk donors and the presence of advanced chronic histologic damage in donor procurement biopsy were strongly associated with moderate-to-severe IFTA. As donor quality is a major risk factor in the development of severe IFTA [14, 16], it was critical to adjust the risk of moderate-to-severe IFTA and other important secondary outcomes for baseline imbalance [40]. Some univariate analyses related to IFTA, and intragraft mRNA gene expression in particular, may have put the valganciclovir group at a disadvantage. Another limitation was the small number of CMV D+/R- patients which does not allow to extrapolate our results to high-risk patients. Generally, the sample size of our study precludes adequate assessment of any potential differences in some secondary outcomes.

## Conclusion

Compared with valacyclovir, valganciclovir prophylaxis is associated with lower risk of moderate-to-severe IFTA at 3 years after renal transplantation. Further studies are warranted to determine whether the more favorable late histologic findings reported with valganciclovir-based regimens stand out only when compared with high-dose valacyclovir prophylaxis or, possibly, with investigational drugs for CMV prevention such as brincidofovir or letermovir [25, 41, 42].

## Additional file

Additional file 1: **Table S1.** Selected genes for intrarenal mRNA gene expression analysis in protocol biopsy at 36 months after transplantation; **Table S2.** Characteristics of the patients with late protocol biopsy; **Table S3.** Maintenance immunosuppressive therapy during the study; **Table S4.** Intrarenal mRNA gene expression in protocol biopsy at 36 months after transplantation according to presence of IFTA. (DOCX 40 kb)

## Abbreviations

aOR: adjusted odds ratio
CI: confidence interval
CMV: cytomegalovirus
D: donor
DSA: donor-specific anti-HLA antibodies
IFTA: interstitial fibrosis and tubular atrophy
PCR: polymerase chain reaction
PVAN: polyomavirus-associated nephropathy
R: recipient

## References

[1] Fishman JA (2017). Infection in organ transplantation. Am J Transplant.

[2] Kotton CN, Kumar D, Caliendo AM, Asberg A, Chou S, Danziger-Isakov L, Humar A (2013). Transplantation society international CMVCG: updated international consensus guidelines on the management of cytomegalovirus in solid-organ transplantation. Transplantation.

[3] Sagedal S, Hartmann A, Nordal KP, Osnes K, Leivestad T, Foss A, Degre M, Fauchald P, Rollag H (2004). Impact of early cytomegalovirus infection and disease on long-term recipient and kidney graft survival. Kidney Int.

[4] Reischig T, Kacer M, Hruba P, Jindra P, Hes O, Lysak D, Bouda M, Viklicky O (2017). The impact of viral load and time to onset of cytomegalovirus replication on long-term graft survival after kidney transplantation. Antivir Ther.

[5] Lollinga WT, Rurenga-Gard L, van Doesum W, van Bergen R, Diepstra A, Vonk JM, Riezebos-Brilman A, Niesters HGM, van Son WJ, van den Born J (2017). High human cytomegalovirus DNAemia early post-transplantation associates with irreversible and progressive loss of renal function - a retrospective study. Transpl Int.

[6] Kaminski H, Fishman JA (2016). The cell biology of cytomegalovirus: implications for transplantation. Am J Transplant.

[7] Dzabic M, Rahbar A, Yaiw KC, Naghibi M, Religa P, Fellstrom B, Larsson E, Soderberg-Naucler C (2011). Intragraft cytomegalovirus protein expression is associated with reduced renal allograft survival. Clin Infect Dis.

[8] Bachelet T, Couzi L, Pitard V, Sicard X, Rigothier C, Lepreux S, Moreau JF, Taupin JL, Merville P, Dechanet-Merville J (2014). Cytomegalovirus-responsive gammadelta T cells: novel effector cells in antibody-mediated kidney allograft microcirculation lesions. J Am Soc Nephrol.

[9] Heutinck KM, Yong SL, Tonneijck L, van den Heuvel H, van der Weerd NC, van der Pant KA, Bemelman FJ, Claas FH, Ten Berge IJ (2016). Virus-specific CD8(+) T cells cross-reactive to donor-alloantigen are transiently present in the circulation of kidney transplant recipients infected with CMV and/or EBV. Am J Transplant.

[10] Reischig T, Jindra P, Svecova M, Kormunda S, Opatrny K, Treska V (2006). The impact of cytomegalovirus disease and asymptomatic infection on acute renal allograft rejection. J Clin Virol.

[11] Courivaud C, Bamoulid J, Chalopin JM, Gaiffe E, Tiberghien P, Saas P, Ducloux D (2013). Cytomegalovirus exposure and cardiovascular disease in kidney transplant recipients. J Infect Dis.

[12] Hjelmesaeth J, Sagedal S, Hartmann A, Rollag H, Egeland T, Hagen M, Nordal KP, Jenssen T (2004). Asymptomatic cytomegalovirus infection is associated with increased risk of new-onset diabetes mellitus and impaired insulin release after renal transplantation. Diabetologia.

[13] Courivaud C, Bamoulid J, Gaugler B, Roubiou C, Arregui C, Chalopin JM, Borg C, Tiberghien P, Woronoff-Lemsi MC, Saas P (2012). Cytomegalovirus exposure, immune exhaustion and cancer occurrence in renal transplant recipients. Transpl Int.

[14] Vanhove T, Goldschmeding R, Kuypers D (2017). Kidney fibrosis: origins and interventions. Transplantation.

[15] Gosset C, Viglietti D, Rabant M, Verine J, Aubert O, Glotz D, Legendre C, Taupin JL, Duong Van-Huyen JP, Loupy A (2017). Circulating donor-specific anti-HLA antibodies are a major factor in premature and accelerated allograft fibrosis. Kidney Int.

[16] Cosio FG, El Ters M, Cornell LD, Schinstock CA, Stegall MD (2016). Changing kidney allograft histology early Posttransplant: prognostic implications of 1-year protocol biopsies. Am J Transplant.

[17] Inkinen K, Soots A, Krogerus L, Loginov R, Bruggeman C, Lautenschlager I (2005). Cytomegalovirus enhance expression of growth factors during the development of chronic allograft nephropathy in rats. Transpl Int.

[18] Reischig T, Jindra P, Hes O, Bouda M, Kormunda S, Treska V (2009). Effect of cytomegalovirus viremia on subclinical rejection or interstitial fibrosis and tubular atrophy in protocol biopsy at 3 months in renal allograft recipients managed by preemptive therapy or antiviral prophylaxis. Transplantation.

[19] Smith JM, Corey L, Bittner R, Finn LS, Healey PJ, Davis CL, McDonald RA (2010). Subclinical viremia increases risk for chronic allograft injury in pediatric renal transplantation. J Am Soc Nephrol.

[20] Hodson EM, Ladhani M, Webster AC, Strippoli GF, Craig JC (2013). Antiviral medications for preventing cytomegalovirus disease in solid organ transplant recipients. The Cochrane database of systematic reviews.

[21] Humar A, Lebranchu Y, Vincenti F, Blumberg EA, Punch JD, Limaye AP, Abramowicz D, Jardine AG, Voulgari AT, Ives J (2010). The efficacy and safety of 200 days valganciclovir cytomegalovirus prophylaxis in high-risk kidney transplant recipients. Am J Transplant.

[22] Reischig T, Jindra P, Mares J, Cechura M, Svecova M, Hes O, Opatrny K, Treska V (2005). Valacyclovir for cytomegalovirus prophylaxis reduces the risk of acute renal allograft rejection. Transplantation.

[23] Lowance D, Neumayer HH, Legendre CM, Squifflet JP, Kovarik J, Brennan PJ, Norman D, Mendez R, Keating MR, Coggon GL (1999). Valacyclovir for the prevention of cytomegalovirus disease after renal transplantation. International Valacyclovir cytomegalovirus prophylaxis transplantation study group. N Engl J Med.

[24] Reischig T, Jindra P, Hes O, Svecova M, Klaboch J, Treska V (2008). Valacyclovir prophylaxis versus preemptive valganciclovir therapy to prevent cytomegalovirus disease after renal transplantation. Am J Transplant.

[25] Reischig T, Hribova P, Jindra P, Hes O, Bouda M, Treska V, Viklicky O (2012). Long-term outcomes of pre-emptive valganciclovir compared with valacyclovir prophylaxis for prevention of cytomegalovirus in renal transplantation. J Am Soc Nephrol.

[26] Reischig T, Kacer M, Jindra P, Hes O, Lysak D, Bouda M (2015). Randomized trial of valganciclovir versus valacyclovir prophylaxis for prevention of cytomegalovirus in renal transplantation. Clinical journal of the American Society of Nephrology : CJASN.

[27] Haas M, Sis B, Racusen LC, Solez K, Glotz D, Colvin RB, Castro MC, David DS, David-Neto E, Bagnasco SM (2014). Banff 2013 meeting report: inclusion of c4d-negative antibody-mediated rejection and antibody-associated arterial lesions. Am J Transplant.

[28] Stegall MD, Park WD, Larson TS, Gloor JM, Cornell LD, Sethi S, Dean PG, Prieto M, Amer H, Textor S (2011). The histology of solitary renal allografts at 1 and 5 years after transplantation. Am J Transplant.

[29] El Ters M, Grande JP, Keddis MT, Rodrigo E, Chopra B, Dean PG, Stegall MD, Cosio FG (2013). Kidney allograft survival after acute rejection, the value of follow-up biopsies. Am J Transplant.

[30] Garcia-Carro C, Dorje C, Asberg A, Midtvedt K, Scott H, Reinholt FP, Holdaas H, Seron D, Reisaeter AV (2017). Inflammation in early kidney allograft surveillance biopsies with and without associated Tubulointerstitial chronic damage as a predictor of fibrosis progression and development of De novo donor specific antibodies. Transplantation.

[31] Modena BD, Kurian SM, Gaber LW, Waalen J, Su AI, Gelbart T, Mondala TS, Head SR, Papp S, Heilman R (2016). Gene expression in biopsies of acute rejection and interstitial fibrosis/tubular atrophy reveals highly shared mechanisms that correlate with worse long-term outcomes. Am J Transplant.

[32] Shimamura M, Seleme MC, Guo L, Saunders U, Schoeb TR, George JF, Britt WJ (2013). Ganciclovir prophylaxis improves late murine cytomegalovirus-induced renal allograft damage. Transplantation.

[33] Battiwalla M, Wu Y, Bajwa RP, Radovic M, Almyroudis NG, Segal BH, Wallace PK, Nakamura R, Padmanabhan S, Hahn T (2007). Ganciclovir inhibits lymphocyte proliferation by impairing DNA synthesis. Biol Blood Marrow Transplant.

[34] Reischig T, Prucha M, Sedlackova L, Lysak D, Jindra P, Bouda M, Matejovic M (2011). Valganciclovir prophylaxis against cytomegalovirus impairs lymphocyte proliferation and activation in renal transplant recipients. Antivir Ther.

[35] Elfadawy N, Flechner SM, Schold JD, Srinivas TR, Poggio E, Fatica R, Avery R, Mossad SB (2014). Transient versus persistent BK viremia and long-term outcomes after kidney and kidney-pancreas transplantation. Clinical journal of the American Society of Nephrology : CJASN.

[36] Sawinski D, Forde KA, Trofe-Clark J, Patel P, Olivera B, Goral S, Bloom RD (2015). Persistent BK viremia does not increase intermediate-term graft loss but is associated with de novo donor-specific antibodies. J Am Soc Nephrol.

[37] Gheith O, Halim MA, Al-Otaibi T, Mansour H, Mosaad A, Atteya HA, Zakaria Z, Said T, Nair P, Nampoory N (2017). Successful cost-effective prevention of cytomegalovirus disease in kidney transplant recipients using low-dose Valganciclovir. Experimental and clinical transplantation : official journal of the Middle East Society for Organ Transplantation.

[38] Schachtner T, Stein M, Babel N, Reinke P (2015). The loss of BKV-specific immunity from Pretransplantation to Posttransplantation identifies kidney transplant recipients at increased risk of BKV replication. Am J Transplant.

[39] Schmidt T, Adam C, Hirsch HH, Janssen MW, Wolf M, Dirks J, Kardas P, Ahlenstiel-Grunow T, Pape L, Rohrer T (2014). BK polyomavirus-specific cellular immune responses are age-dependent and strongly correlate with phases of virus replication. Am J Transplant.

[40] Roberts C, Torgerson DJ (1999). Understanding controlled trials: baseline imbalance in randomised controlled trials. BMJ.

[41] Marty FM, Winston DJ, Rowley SD, Vance E, Papanicolaou GA, Mullane KM, Brundage TM, Robertson AT, Godkin S, Mommeja-Marin H (2013). CMX001 to prevent cytomegalovirus disease in hematopoietic-cell transplantation. N Engl J Med.

[42] Marty FM, Ljungman P, Chemaly RF, Maertens J, Dadwal SS, Duarte RF, Haider S, Ullmann AJ, Katayama Y, Brown J (2017). Letermovir prophylaxis for cytomegalovirus in hematopoietic-cell transplantation. N Engl J Med.

