# Supplementary material for: Less renal allograft fibrosis with valganciclovir prophylaxis for cytomegalovirus compared to high-dose valacyclovir: a parallel group, open-label, randomized controlled trial
Source: BMC Infect Dis. 2018 Nov 15;18:573. doi: 10.1186/s12879-018-3493-y (PMC6238264; doi:10.1186/s12879-018-3493-y)
Supplement: Supplementary file 1 — Table S1. Selected genes for intrarenal mRNA gene expression analysis in protocol biopsy at 36 months after transplantation; Table S2. Characteristics of the patients with late protocol biopsy; Table S3. Maintenance immunosuppressive therapy during the study; Table S4. Intrarenal mRNA gene expression in protocol biopsy at 36 months after transplantation according to presence of IFTA. (DOCX 40 kb) [file 12879_2018_3493_MOESM1_ESM.docx]

**Supplementary Table 1.** Selected genes for intrarenal mRNA gene expression analysis in protocol biopsy at 36 months after transplantation^a^

| **Assay ID** | **Group** | **Gene Symbol** | **Gene name** | **Pathways/function** |
| --- | --- | --- | --- | --- |
| Hs99999905_m1 | Dehydrogenase | GAPDH | glyceraldehyde-3-phosphate dehydrogenase | Endogenous control |
| Hs00237013_m1 | Chemokine | CCL11 | chemokine (C-C motif) ligand 11 | Chemokine Signaling, TGF-Beta Pathway, Transendothelial Migration of Leukocytes, TH2 Cytokines, Chemokine (C-C motif) Ligands |
| Hs00171123_m1 | Chemokine | CCL16 | chemokine (C-C motif) ligand 16 | Chemokine Signaling, TGF-Beta Pathway, Transendothelial Migration of Leukocytes, Chemokine (C-C motif) Ligands |
| Hs00234140_m1 | Chemokine | CCL2 | chemokine (C-C motif) ligand 2 | Chemokine Signaling, Renin-Angiotensin Pathway, TGF-Beta Pathway, Transendothelial Migration of Leukocytes, Chemokine (C-C motif) Ligands |
| Hs00174575_m1 | Chemokine | CCL5 | chemokine (C-C motif) ligand 5 | Chemokine Signaling, TGF-Beta Pathway, Transendothelial Migration of Leukocytes, TH2 Cytokines , Chemokine (C-C motif) Ligands, Inflammatory Response |
| Hs00236937_m1 | Chemokine | CXCL1 | chemokine (C-X-C motif) ligand 1 (melanoma growth stimulating activity, alpha) | Chemokine Signaling, Transendothelial Migration of Leukocytes , Chemokine (C-X-C motif) Ligands, Inflammatory Response |
| Hs00171138_m1 | Cytokine | CXCL11 | chemokine (C-X-C motif) ligand 11 | Chemokine Signaling, Transendothelial Migration of Leukocytes , Chemokine (C-X-C motif) Ligands |
| Hs00601975_m1 | Chemokine | CXCL2 | chemokine (C-X-C motif) ligand 2 | Chemokine Signaling, Transendothelial Migration of Leukocytes , Chemokine (C-X-C motif) Ligands, Inflammatory Response |
| Hs00605742_g1 | Chemokine | CXCL6 | chemokine (C-X-C motif) ligand 6 (granulocyte chemotactic protein 2) | Chemokine Signaling, Transendothelial Migration of Leukocytes , Chemokine (C-X-C motif) Ligands, Inflammatory Response |
| Hs00152917_m1 | G-protein coupled receptor | CCR5 | chemokine (C-C motif) receptor 5 | Chemokine Signaling, Transendothelial Migration of Leukocytes, Genes Involved in T-cell Polarization, Chemokine (C-C motif) Receptors |
| Hs00174843_m1 | G-protein coupled receptor | CXCR6 | chemokine (C-X-C motif) receptor 6 | Chemokine Signaling, Transendothelial Migration of Leukocytes |
| Hs00166223_m1 | Cytokine receptor | IFNGR1 | interferon gamma receptor 1 | Interferon Pathway, Genes Involved in Th1/Th2 Differentiation |
| Hs00233470_m1 | Cytokine | BMP6 | bone morphogenetic protein 6 | Cellular Apoptosis Pathway, mTOR Pathway, Renin-Angiotensin Pathway, TGF-Beta Pathway |
| Hs00167060_m1 | Cytokine | GDF5 | growth differentiation factor 5 | Cellular Apoptosis Pathway, Renin-Angiotensin Pathway |
| Hs00170103_m1 | Cytokine | INHBA | inhibin, beta A | Macrophage Activation, T-cell Activation, TGF-b Superfamily Cytokines |
| Hs00386448_m1 | Hsp 70 family chaperone | LTBP1 | latent transforming growth factor beta binding protein 1 | Cellular Apoptosis Pathway, Renin-Angiotensin Pathway, TGF-Beta Pathway, TGF-b Superfamily Cytokines, Extracellular Matrix Structural Constituents |
| Hs00166367_m1 | Hsp 70 family chaperone | LTBP2 | latent transforming growth factor beta binding protein 2 | Cellular Apoptosis Pathway, Renin-Angiotensin Pathway, TGF-Beta Pathway, TGF-b Superfamily Cytokines, Extracellular Matrix Structural Constituents |
| Hs00186025_m1 | Hsp 70 family chaperone | LTBP4 | latent transforming growth factor beta binding protein 4 | Cellular Apoptosis Pathway, Renin-Angiotensin Pathway, TGF-Beta Pathway, TGF-b Superfamily Cytokines, Extracellular Matrix Structural Constituents |
| Hs00234042_m1 | Growth factor | PDGFB | platelet-derived growth factor beta polypeptide (simian sarcoma viral (v-sis) oncogene homolog) | Cellular Apoptosis Pathway, Renin-Angiotensin Pathway, TGF-b Superfamily Cytokines, Other Extracellular Molecules |
| Hs99999918_m1 | Cytokine | TGFB1 | transforming growth factor, beta 1 | Cellular Apoptosis Pathway, IL-2 Gene Expression in Activated and Quiescent T-Cells, Renin-Angiotensin Pathway, TGF-Beta Pathway, Genes Involved in T-cell Polarization |
| Hs00234244_m1 | Cytokine | TGFB2 | transforming growth factor, beta 2 | Cellular Apoptosis Pathway, IL-2 Gene Expression in Activated and Quiescent T-Cells, Renin-Angiotensin Pathway, TGF-Beta Pathway |
| Hs00170630_m1 | Other transcription factor | FOS | v-fos FBJ murine osteosarcoma viral oncogene homolog | Chemokine Signaling, Renin-Angiotensin Pathway, TGF-Beta Pathway, TNF Superfamily Pathway, Inflammatory Response, SMAD Target Genes |
| Hs00164004_m1 | Protein kinase | COL1A1 | collagen, type I, alpha 1 | Transendothelial Migration of Leukocytes, SMAD Target Genes |
| Hs00164099_m1 | Protein kinase | COL1A2 | collagen, type I, alpha 2 | Transendothelial Migration of Leukocytes, SMAD Target Genes |
| Hs00164103_m1 | Protein kinase | COL3A1 | collagen, type III, alpha 1 | Transendothelial Migration of Leukocytes, SMAD Target Genes |
| Hs00357891_s1 | Other transcription factor | JUNB | jun B proto-oncogene | Interferon Pathway, SMAD Target Genes, Other Transcription Factors |
| Hs00232222_m1 | Other transcription factor | SMAD3 | SMAD family member 3 | IL-2 Gene Expression in Activated and Quiescent T-Cells, TGF-Beta Pathway |
| Hs00232068_m1 | Other transcription factor | SMAD4 | SMAD family member 4 | IL-2 Gene Expression in Activated and Quiescent T-Cells, TGF-Beta Pathway |
| Hs00195437_m1 | Other transcription factor | SMAD5 | SMAD family member 5 | TGF-Beta Pathway |
| Hs00195441_m1 | Other transcription factor | SMAD9 | SMAD family member 9 | TGF-Beta Pathway |
| Hs01567025_m1 | Membrane-bound signaling molecule | CD86 | CD86 molecule | CTLA4 Signaling, Regulators of T-cell Activation |
| Hs00231733_m1 | Transcription cofactor | CREBBP | CREB binding protein | NF-KappaB Family Pathway, TGF-Beta Pathway, CD4+T Cell Marker |
| Hs00232342_m1 | Other transcription factor | NFATC1 | nuclear factor of activated T-cells, cytoplasmic, calcineurin-dependent 1 | IL-2 Gene Expression in Activated and Quiescent T-Cells, TH2 Cytokines, VEGF Signaling Pathway |
| Hs00234855_m1 | Other transcription factor | NFATC2 | nuclear factor of activated T-cells, cytoplasmic, calcineurin-dependent 2 | IL-2 Gene Expression in Activated and Quiescent T-Cells, TH2 Cytokines, VEGF Signaling Pathway |
| Hs00765730_m1 | Other transcription factor | NFKB1 | nuclear factor of kappa light polypeptide gene enhancer in B-cells 1 | Cellular Apoptosis Pathway, Chemokine Signaling, IL-2 Gene Expression in Activated and Quiescent T-Cells, NF-KappaB Family Pathway, Renin-Angiotensin Pathway, Inflammatory Response |
| Hs00234829_m1 | Other transcription factor | STAT1 | signal transducer and activator of transcription 1 | Interferon Pathway, Renin-Angiotensin Pathway, TH1 Cytokines |
| Hs00237139_m1 | Miscellaneous function | STAT2 | signal transducer and activator of transcription 2 | Interferon Pathway, JAK / STAT Signaling Pathway |
| Hs00374280_m1 | Other transcription factor | STAT3 | signal transducer and activator of transcription 3 (acute-phase response factor) | Renin-Angiotensin Pathway, Transcriptional Regulator |
| Hs00231372_m1 | Other transcription factor | STAT4 | signal transducer and activator of transcription 4 | TH1 Cytokines |
| Hs00273500_m1 | Other transcription factor | STAT5B | signal transducer and activator of transcription 5B | Chemokine Signaling, Cytokine and Chemokine-mediated Signaling Pathways |
| Hs00153340_m1 | Other transcription factor | TP53 | tumor protein p53 | Cellular Apoptosis Pathway, p53 and DNA Damage Response, Wnt Signaling Pathway |
| Hs00204833_m1 | RIG–I-like receptor family | DDX58 | DEAD (Asp-Glu-Ala-Asp) Box Polypeptide 58 | NF-kappa B signaling pathway, RIG-I-like receptor signaling pathway, Cytosolic DNA-sensing pathway |
| Hs00164932_m1 | Membrane-bound signaling molecule | ICAM1 | Intercellular adhesion molecule 1 | Natural killer cell mediated cytotoxicity, Cell adhesion molecules, TNF signaling pathway, Leukocyte transendothelial migration, NF-kappa B signaling pathway |
| Hs01070332_m1 | RIG–I-like receptor family | IFIH1 | Interferon induced with helicase C domain 1 | Defence response, immune response, RIG-I-like receptor signaling pathway |
| Hs01551078-m1 | Toll-like receptor family | TLR3 | Toll-like receptor 3 | Toll-like receptor signaling pathway |
| Hs00394497_m1 | Intracellular serpins | SERPINB9 | Serpin Peptidase Inhibitor, Clade B (Ovalbumin), Member 9 | Cellular Apoptosis Pathway |

^a^Small portions of renal tissue from the cortical zone were immediately stored in a preservation solution for expression analysis and RT-qPCR was performed as described previously.[^1^](#_ENREF_1)^,^[^2^](#_ENREF_2) The selection of 40 genes were based on significant association with interstitial fibrosis and tubular atrophy, CMV viremia status and/or CMV prevention mode in our previous study[^1^](#_ENREF_1), while 5 additional genes related to antiviral response were selected according to literature.[^3-6^](#_ENREF_3)

**References**

1. Reischig T, Hribova P, Jindra P, et al. Long-term outcomes of pre-emptive valganciclovir compared with valacyclovir prophylaxis for prevention of cytomegalovirus in renal transplantation. *J Am Soc Nephrol.* 2012;23(9):1588-1597.

2. Reischig T, Kacer M, Hruba P, et al. The impact of viral load and time to onset of cytomegalovirus replication on long-term graft survival after kidney transplantation. *Antivir Ther.* 2017;22(6):503-513.

3. Heutinck KM, Rowshani AT, Kassies J, et al. Viral double-stranded RNA sensors induce antiviral, pro-inflammatory, and pro-apoptotic responses in human renal tubular epithelial cells. *Kidney Int.* 2012;82(6):664-675.

4. Heutinck KM, Kassies J, Florquin S, ten Berge IJ, Hamann J, Rowshani AT. SerpinB9 expression in human renal tubular epithelial cells is induced by triggering of the viral dsRNA sensors TLR3, MDA5 and RIG-I. *Nephrol Dial Transplant.* 2012;27(7):2746-2754.

5. Sester U, Presser D, Dirks J, Gartner BC, Kohler H, Sester M. PD-1 expression and IL-2 loss of cytomegalovirus- specific T cells correlates with viremia and reversible functional anergy. *Am J Transplant.* 2008;8(7):1486-1497.

6. Li Y, Yan H, Xue WJ, et al. Allograft rejection-related gene expression in the endothelial cells of renal transplantation recipients after cytomegalovirus infection. *Journal of Zhejiang University Science B.* 2009;10(11):820-828.

**Supplementary Table 2.** Characteristics of the patients with late protocol biopsy

| Characteristic | Valganciclovir  (n = 51) | Valacyclovir  (n = 50) | *P*  Value |
| --- | --- | --- | --- |
| Recipient |  |  |  |
| Age (yr) | 48 ± 13 | 49 ± 11 | 0.5 |
| Gender (male) | 39 (76) | 33 (66) | 0.3 |
| Previous transplantation | 9 (15) | 7 (12) | 0.8 |
| Renal replacement therapy duration (mo) | 18 ± 16 | 24 ± 22 | 0.1 |
| HLA mismatches (n) | 3.5 ± 1.2 | 3.6 ± 1.5 | 0.5 |
| Pretransplant PRA ≥20% | 9 (18) | 7 (14) | 0.8 |
| CMV serostatus |  |  | 0.8 |
| D+/R- | 4 (8) | 3 (6) |  |
| D+/R+ | 41 (80) | 42 (84) |  |
| D-/R+ | 6 (12) | 5 (10) |  |
| Donor |  |  |  |
| Age (yr) | 50 ± 16 | 48 ± 16 | 0.6 |
| Donor type (deceased) | 48 (94) | 47 (94) | 0.7 |
| Expanded criteria donor^a^ | 28 (55) | 27 (54) | 0.9 |
| Donor after cardiac death | 3 (6) | 3 (6) | 1.0 |
| Dual kidney transplantation | 4 (8) | 3 (6) | 1.0 |
| Advanced chronic histologic damage^b^ | 11 (22) | 7 (14) | 0.3 |
| Primary immunosuppression |  |  |  |
| Cyclosporine + mycophenolate mofetil | 23 (45) | 31 (62) | 0.09 |
| Tacrolimus + mycophenolate mofetil | 28 (55) | 19 (38) |  |
| No induction therapy | 23 (45) | 30 (60) | 0.1 |
| Basiliximab | 21 (41) | 11 (22) | 0.04 |
| Thymoglobulin | 7 (13) | 9 (18) | 0.8 |

Data are number of patients (percentage) or mean ± SD. CMV, cytomegalovirus; D, donor; PRA, panel reactive antibodies; R, recipient.

^a^According to the United Network for Organ Sharing criteria.

^b^A minimum 1 of the following findings on donor procurement biopsy: moderate-to-severe vascular nephrosclerosis, diabetic nephropathy, and/or ≥15% of glomerulosclerosis. Procurement biopsy was performed in 54 selected donors considered to be at increased risk.

**Supplementary Table 3.** Maintenance immunosuppressive therapy during the study

| Characteristic | Valganciclovir  (n = 60) | Valacyclovir  (n = 59) | *P*  Value |
| --- | --- | --- | --- |
| Month 12^a^ |  |  |  |
| Tacrolimus | 44 (77) | 43 (80) | 0.9 |
| Cyclosporine | 13 (23) | 11 (20) |  |
| Sirolimus | 1 (2) | 1 (2) | 0.5 |
| Mycophenolate mofetil | 54 (95) | 50 (93) | 0.9 |
| Prednisone | 55 (96) | 51 (94) | 0.9 |
| Month 24^a^ |  |  |  |
| Tacrolimus | 44 (79) | 44 (81) | 0.9 |
| Cyclosporine | 11 (20) | 9 (17) | 0.9 |
| Sirolimus | 1 (2) | 1 (2) | 0.5 |
| Mycophenolate mofetil | 46 (82) | 51 (94) | 0.09 |
| Prednisone | 54 (96) | 49 (91) | 0.4 |
| Month 36^a^ |  |  |  |
| Tacrolimus | 44 (81) | 44 (81) | 0.8 |
| Cyclosporine | 10 (19) | 8 (15) | 0.8 |
| Sirolimus | 0 (0) | 2 (4) | 0.5 |
| Mycophenolate mofetil | 51 (94) | 51 (94) | 0.7 |
| Prednisone | 53 (98) | 50 (93) | 0.4 |
| Tacrolimus trough level (ng/mL) |  |  |  |
| Month 12 | 6.3 ± 2.3 | 6.5 ± 2.1 | 0.6 |
| Month 24 | 6.1 ± 1.7 | 6.3 ± 1.7 | 0.6 |
| Month 36 | 6.2 ± 1.7 | 5.8 ± 1.6 | 0.2 |
| Cyclosporine trough level (ng/mL) |  |  |  |
| Month 12 | 117 ± 21 | 118 ± 49 | 0.9 |
| Month 24 | 116 ± 37 | 98 ± 24 | 0.2 |
| Month 36 | 103 ± 39 | 89 ± 27 | 0.4 |
| Mycophenolate mofetil dose (g per day) |  |  |  |
| Month 12 | 1.25 ± 0.56 | 1.34 ± 0.58 | 0.5 |
| Month 24 | 1.26 ± 0.59 | 1.29 ± 0.55 | 0.9 |
| Month 36 | 1.27 ± 0.59 | 1.21 ± 0.51 | 0.7 |
| Mycophenolic acid AUC (mg*h/L) |  |  |  |
| Month 36 | 42 ± 16 | 43 ± 13 | 0.7 |
| Prednisone dose (mg per day) |  |  |  |
| Month 12 | 5 ± 1 | 5 ± 1 | 0.9 |
| Month 24 | 5 ± 0 | 5 ± 1 | 0.9 |
| Month 36 | 5 ± 0 | 5 ± 0 | 0.9 |

Data are number of patients (percentage) or mean ± SD. AUC, area under concentration curve.

^a^Assessed in patients with functioning graft.

**Supplementary Table 4.** Intrarenal mRNA gene expression in protocol biopsy at 36 months after transplantation according to presence of IFTA^a^

| Gene Symbol | Moderate-to-Severe IFTA  (n = 25) | No IFTA or Mild IFTA without Inflammation  (n = 50) | *P*  Value^b^ |
| --- | --- | --- | --- |
| CCL2 | 1.46 (0.51-2.15) | 0.41 (0.23-1.00) | 0.003 |
| CCL5 | 8.07 (5.00-27.2) | 3.01 (1.49-6.92) | 0.004 |
| CCL11 | 1.28 (0.61-1.79) | 0.30 (0.18-0.62) | 0.001 |
| CCL16 | 2.40 (0.34-3.77) | 5.33 (2.83-7.51) | 0.005 |
| CCR5 | 3.75 (1.59-8.00) | 0.95 (0.50-2.91) | <0.001 |
| CD86 | 1.35 (0.68-3.11) | 0.53 (0.22-1.04) | 0.007 |
| CXCL1 | 0.28 (0.20-0.86) | 0.06 (0.03-0.16) | <0.001 |
| CXCL2 | 0.05 (0.02-0.07) | 0.02 (0.01-0.03) | <0.001 |
| CXCL6 | 2.34 (1.39-7.22) | 0.56 (0.23-1.02) | <0.001 |
| CXCL11 | 9.42 (2.15-23.8) | 1.46 (0.53-5.53) | 0.002 |
| CXCR6 | 1.78 (1.05-6.27) | 0.59 (0.40-2.52) | 0.01 |
| FOS | 1.19 (0.68-1.96) | 0.70 (0.37-1.08) | 0.02 |
| INHβA | 2.94 (1.08-7.85) | 0.93 (0.52-1.91) | 0.01 |
| JUNB | 0.86 (0.46-1.93) | 0.54 (0.34-0.93) | 0.04 |
| STAT1 | 1.94 (0.85-3.93) | 1.00 (0.51-2.11) | 0.04 |
| STAT4 | 8.50 (4.42-16.4) | 3.37 (1.58-7.44) | 0.003 |
| TGFβ1 | 1.59 (0.76-2.97) | 0.85 (0.59-1.77) | 0.03 |
| TGFβ2 | 1.39 (0.86-2.39) | 0.88 (0.55-1.45) | 0.03 |
| ICAM1 | 2.67 (0.97-4.17) | 0.85 (0.59-1.86) | 0.01 |
| IFIH1 | 1.37 (0.87-2.47) | 0.64 (0.42-1.03) | <0.001 |
| SERPINB9 | 4.26 (2.79-7.35) | 2.01 (1.33-3.50) | 0.005 |

Data are median and interquartile range. Results are expressed as the ratio of the gene of interest to the housekeeping GAPDH gene. For complete names and functional characteristics of genes, see Supplementary Table 1. IFTA, interstitial fibrosis and tubular atrophy.

^a^All genes with a statistically significant difference are shown. Because of insufficient material or RNA extraction failure, intrarenal mRNA gene expression analysis could be performed in 40 (valganciclovir group) and 45 (valacyclovir group) biopsies, respectively.

^b^Mann-Whitney U-test.
